# Tinea capitis and its associated factors among school children in Gondar town northwest, Ethiopia

**DOI:** 10.1186/s12887-024-04917-6

**Published:** 2024-07-12

**Authors:** Tewodros Getaneh Alemu, Nigus Getaneh Alemu, Almaz Tefera Gonete

**Affiliations:** 1 Department of Pediatrics and Child Health Nursing, School of Nursing, College of Medicine and Health Sciences, University of Gondar, P.O.Box: 196, Gondar, Ethiopia; 2 School of Medicine, College of Medicine and Health Sciences, University of Gondar, Gondar, Ethiopia

## Abstract

**Introduction:**

Tinea capitis is a global public health concern with a unique therapeutic challenge and mostly affects children. The burden is double in developing countries. There is no study on school-going children from the urban residence in Ethiopia.

**Objective:**

To determine the prevalence of Tinea capitis and its associated factors among school children in Gondar town northwest, Ethiopia 2021.

**Methods:**

An institution-based cross-sectional study was conducted among school children in Gondar town from November 20 to December 20, 2021. Data was collected through an interviewer-administered structured questionnaire. A stratified simple random sampling technique was employed. Then data were entered into EPI Info version 7.22 and transferred to Statistical package for social science (SPSS) version 22 for further analysis. The binary logistic regression model was employed to identify factors associated with tinea capitis, and the strength of association and statistical significance was declared using the adjusted odds ratios with its corresponding 95% CI, and p-value ≤ 0.05 respectively.

**Result:**

The prevalence of tinea capitis among school children in Gondar town was found to be 29.4%. Public school student (AOR = 2.79 95% CI: 1.34–5.87), widowed mother (AOR = 6.09, 95% CI: 1.83–11.23), students in the age group of 5–8 years (AOR = 3.79 95% CI: 1.68–8.55), animal contact (AOR = 2.61 95% CI: 1.15–5.90), and family similar illness category (AOR = 8.49 95% CI: 3.73–11.39) have risen the likelihood of tinea capitis among school children.

**Conclusions:**

The prevalence of tinea capitis was higher. Young age, children from widowed marital status, illiterate mother, history of share blades, animal contact, a family similar illness, and lower number of living rooms are important factors contributing to tinea capitis among school children. Health education for the mother on the mode of transmission, prevention, and improve the low socioeconomic status of the parent is crucial.

**Key terms:**

Children, Ethiopia, Gondar, School age, Tinea capitis

## Introduction

Tinea capitis is a fungal infection that affects the skin of the scalp, brows, and eyelashes, with a tendency for attacking hair shafts and follicles. Tinea capitis, also known as “herpes tonsurans ringworm of the hair, ringworm of the scalp, and Tinea tonsurans, is a dermatophytosis (scalp fungal infection) [1]. The organisms that cause Tinea capitis are typically found as fomites on objects like pillows, combs, hats, and theater seats, where the spores can survive for a very long time and facilitate in the disease’s spread [2]. Globally Tinea capitis, a dermatophytosis of the scalp that typically affects youngsters and makes up to 92.5% of all dermatophytoses in children under the age of ten, is a public health burden with a distinct therapeutic challenges [3]. Fungal infections are primarily seen in tropical and low-income nations. Fungal skin diseases are a major issue only in Sub-Saharan Africa (SSA) [4, 5].

Its prevalence has continued to have a dramatic increase in the last decades with more than 20–25% of the world’s population being affected [6], which cause serious morbidity and mortality around the globe, resulting in the death of 1.5 to 2.0 million people annually [7]. Several studies conducted in developing nations have shown that school-age children suffer from a high prevalence of skin disorders, including superficial fungal infections (Tinea capitis) and a wide spectrum of other infectious dermatoses [8, 9]. Ten to thirty% of school-age children in Africa have Tinea infection, making it one of the most affected regions [10]. Around one in five school children in Africa has Tinea capitis making it one of the most common childhood conditions in the region, Ethiopia is the second country affected by Tinea capitis in the continent [11]. Similarly, in Ethiopia, the prevalence of Tinea capitis ranges from 20 − 47% among school-aged children [12].

When a child experiences severe hair loss from Tinea capitis, the long-term detrimental effects of the illness can include depression symptoms, emotional distress, social anxiety, low self-esteem, and suicide thoughts. For women in particular, the burden of these effects is double that of men in later life [13, 14]. The psychosocial impact of Tinea capitis in school children is more serious in the daily lives of school children [15, 16]. Children affected by these problems may find it difficult to focus on their education on a regular basis, which could result in dropout rates [17].

Factors that contribute to Tinea capitis are sharing facilities, being male, having poor personal hygiene, close contact to others who have the disease, being overcrowded, having low socioeconomic status, and parents with different low of education and occupation [18].

Although their substantial impact on the international death toll, fungal diseases continue to be ignored in mainstream health programming and masking the true magnitude of fungal disease burden and its socioeconomic effect influence its prioritization within the globalhealth outline [19]. Despite few studies done on Tania capitis in Ethiopia; there is a huge research gap on Tinea capitis in school children in Ethiopia Furthermore, the findings of this study contribute to battling Tinea capitis among this segment of the population through providing evidence to the concerned bodies to give intervention. Therefore, this study aims to determine the prevalence of Tinea capitis and its associated factor among school children in Gondar city, Northwest Ethiopia, 2021.

## Method

### Study design and period

An institution based cross-sectional study was conducted from November 20 to December 20, 2021 G.C.

### Study area

Gondar town is found 748 km far away from Addis Ababa. Gondar is the fourth largest city in Ethiopia with a population of 358,257 people according to Gondar town statistics office. In the town, there are forty-three private and public primary schools and 16,331 students in the 2020/2021 academic year, Gondar is the city in the central Gondar zone which is in the Amhara region located in the Northwest part of Ethiopia.

### Population

#### Source population

School going children in Gondar town registered during the academic year 2021.

#### Study population

All children randomly selected from schools of Gondar town and available during the data collection period were the study population.

#### Inclusion and exclusion criteria

This study includes Gondar town’s children who are in school. However, children who have a medical diagnosis of mental or physical illness and residing in the study area for less than six months were excluded from the study.

#### Sample size determination

Sample size was determined using single population proportion formula considering the following assumption: 95% confidence level (Za/2), 5% margin of error (d) and proportion (p) of Tinea capitis in previous study was taken as 47.5% [12].

$$n\left( {sample\,size} \right) = {\left( {{{Z\alpha } \mathord{\left/{\vphantom {{Z\alpha } 2}} \right.\kern-\nulldelimiterspace} 2}} \right)^2}p{{\left[ {1 - p} \right]} \mathord{\left/{\vphantom {{\left[ {1 - p} \right]} {{d^2}}}} \right.\kern-\nulldelimiterspace} {{d^2}}}$$

*n* = 383 and the final sample size became 402 with 5% non-response rate.

#### Sampling technique and procedure

Initially, all primary schools in Gondar town were listed. Simple random sampling method (lottery) was used to select two private and four public schools. The student’s classroom register was used as a sampling frame. All sections were separately enlisted and 20% of section from each grade was selected by lottery method. Proportional allocation method was done to select the number of students participated from the selected school, grade and section.

### Variable of the study

#### Dependent variable

Tinea capitis (yes/no).

#### Independent variable

##### Socio demographic variables

Age, sex, religion, place of residence, family size, education of child, education of mother, education of father, occupation of father, occupation of mother, number of living room, family income.

##### Tinea capitis related variables

Itching, loss of hair, scarring alopecia, oozing of the lesion, scaly lesion, sharing of bed, sharing of blades/scissors for cutting hair, history of contact with animals, presence Tinea capitis in the family member.

#### Operational definition

##### Tinea capitis

Clinical diagnose was made in children who had one or more signs associated with Tinea capitis (hair loss, dry scaly areas, redness, and itch) [20].

### Data collection tool and procedure

We collected our data using interviewer administrated questionnaire. Physical examination was also done for all subjects. The tool comprised of information on demographic, socio-economic status, and history of contact with animals which are gathered by reviewing previous studies and reports. Adequate information about the procedures was given to the selected participants. Following the information given to the selected children, just a few questions were forwarded to their families to respond to at home. The selected students were interviewed in the school outside of the classroom. General physician examined each individual regarding symptom, and the physical examination was also filled out in the same questionnaire. Furthermore, about six nurse were recruited as data collectors and two supervisor who are medical doctor were recruited as a supervisor.

### Data quality control

The questionnaire was prepared in English and then it was translated in to Amharic language. The question administered by the interview during data collection for each study subjects. It was reviewed by dermatologist and public health experts to check its appropriateness for assessing Tinea capitis. As well as some independent factors like: loss of hair, scarring alopecia, oozing of the lesion, and scaly lesion. Data collectors and supervisors were trained for one day regarding technique, ethical issue and data collection process by the principal investigator before the actual data collection.

### Data processing and analysis

Data was cleaned, coded and entered using EPI info version 7.22 and exported to SPSS version 22 statistical packages for further statistical analyses. Descriptive statistics was used to describe the socio-demographic characteristics of the respondents, by using texts, tables and graphs. Binary logistic regression analysis was applied to identify factors associated with Tinea capitis. Bivariable analysis was done and P value less than 0.2 was included in the multiple logistic regression analysis. Multivariable logistic regression analysis was employed to identify factors associated with Tinea capitis by controlling the effect of potential confounding variables. The Hosmer and Lemeshow test were used to diagnose the model adequacy. Finally, variables which had independent association with Tinea capitis was identified on the basis of Adjusted Odds Ratio and p-value with its corresponding 95% CI. Variables having a p-value less than 0.05 was claimed as statistically significant.

### Ethical consideration

Ethical clearance was obtained from College of Medicine and Health Sciences, School of Nursing on the behalf of the Institutional Review Board (IRB) of University of Gondar. Then, a permission letter written from the Gondar education office to the selected schools. Informed consent obtained from all the participants parents/guardians. After informing them all the purpose, benefit, risk, the confidentiality of the information and the voluntary nature of the participation in the study. Then assent was obtained from the children. So, participation was after obtaining both informed consent and assent. Participation was on a voluntary basis and confidentiality was maintained to encourage accurate and honest self-disclosure.

## Result

### Socio-demographic characteristics

A total of 402 school-age children participated in the survey, with a response rate of 98% (394) and 41.6% of them were female. Among the students attending school, half (50.8%) were in grades 1–4 and of the school-age children, one-fourth (25.4%) belonged to the 5–8 age group. Their mean age was 10.4 (± 2.29 SD). The majority of participants (76.4.5%) identify as Orthodox; the remaining participants (17.5%) and 6.1%) are Muslims and Protestants, respectively. Over 52.5% of the participants are part of families with five or more members (Table 1).

**Table 1:** Socio-demographic characteristics among school children in Gondar city, 2021 (*n* = 394)

Variables	Response	Frequency	Percent
Age	5–8	100	25.4%
	9–11	142	36.2%
	12–15	152	38.4%
Sex	Female	164	41.6%
	Male	230	58.4%
Type of school	Private	154	39.1%
	Public	240	60.9%
Educational level	1–4	200	50.8%
	5–8	194	49.2%
Residence	urban	393	99.7%
	Rural	1	0.3%
Religion	Orthodox	301	76.4%
	Muslim	69	17.5%
	Protestant	24	6.1%
Marital status	Married	321	81.5%
	Single	28	7.1%
	Divorced	19	4.8%
	Windowed	26	6.6%
Education of the father	Unable to read and write	25	6.3%
	Read and write	90	22.8%
	Primary school	53	13.5%
	Secondary school	84	21.3%
	College and above	142	36%
Occupation of the father	Government employee	159	40.4%
	Farmer	21	5.3%
	Daily labor	36	9.1%
	Merchant	178	45.2%
Education of the mother	Unable to read and write	15	3.8%
	Read and write	116	29.5%
	Primary school	59	15.0%
	Secondary school	60	15.2%
	College and above	144	36.6%
Occupation of the mother	Housewife	149	37.8%
	Government employee	112	28.4%
	Farmer	2	0.5%
	Daily labor	43	10.9%
	Merchant	88	22.3%
Family size	1–4	187	47.5%
	5 and above	207	52.5%
Living room	1–2	166	42.1%
	3 and above	228	57.9%

### Risk factors and signs related to Tinea capitis of school children

Majority (76.9%) of school children had history of animal contact. Sixty-six (16.8%) of them had a family history of a similar illness. Schoolchildren share beds in 78.2% of cases. A scaly lesion affected nearly one-third (29.9%) of school-aged children (Table 2).

**Table 2:** Risk factors and signs related to Tinea capitis among school children in Gondar city, 2021 (*n* = 394)

Variables	Response	Frequency	Percent
Iching hair	Yes	113	28.7%
	No	281	71.3%
Hair loss	Yes	95	24.1%
	No	299	75.9%
Alopecia	Yes	27	6.9%
	No	367	93.1%
Oozing lesion	Yes	65	16.5%
	No	329	83.5%
Scaly lesion	Yes	118	29.9%
	No	276	70.1%
Share beds	Yes	308	78.2%
	No	86	21.8%
Share blades	Yes	143	36.3%
	No	251	63.7%
Animal contact	Yes	303	76.9%
	No	91	23.1%
Family similar illness	Yes	66	16.8%
	No	328	83.2%

### Prevalence of Tinea capitis among school children in Gondar city

The prevalence of Tinea capitis among school children in Gondar city was found to be 29.4% with 95% CI (24.9-34.0%).

### Factors associated with Tinea capitis among school children

Bi-variable analysis was carried out and variable associated in the Bi-variable analysis were age, type of school, level of education, religion, marital status, education of the father, occupation of the father, education of the mother, share blades, share beds, animal contact, family history of similar illness, family size, and living room. In multivariable analysis, eight of them were found to be significantly associated with Tinea capitis such as: age, type of school, marital status, education of the mother, share blades, animal contact, family history of similar illness, and living room. The risk of developing Tinea capitis in children of widowed mothers is six times higher than that of children of married mothers (AOR = 6.09, 95% CI: 1.83–11.23). Children who were in the 5–8 age category were 3.8 times more likely to have Tinea capitis as compared to children in the 12–15 age category (AOR = 3.79 95% CI:1.68–8.55). Children who had history of animal contact are 2.6 times higher to develop Tinea capitis compared to children who had no history of animal contact (AOR = 2.61 95% CI:1.15–5.90) (Table 3).

**Table 3:** Bivariate and multivariable logistic regression of factors associated with tinea capitis among school children in Gondar city, 2021 (*n* = 394)

Variables	Response	Tinea capitis		COR(95% CI)	AOR(95% CI)
		No	Yes		
Age	5–8	64	36	1.81(1.04–3.15)	3.79(1.68–8.55)***
	9–11	98	44	1.45(0.86–2.42)	0.51(0.20–3.25)
	12–15	116	36	1	1
Type of school	Private	130	24	1	1
	Public	148	92	3.37(2.03–5.59)	2.79(1.34–5.87)***
Level of education	1–4	131	69	1.65(1.06–2.56)	1.58(0.54–4.58)
	5–8	147	47	1	1
Religion	Orthodox	209	92	2.56(1.31–7.12)	3.58(0.40–3.81)
	Muslim	46	23	2.01(1.56–9.63)	1.51(0.15–6.35)
	Protestant	18	6	1	1
Marital status	Married	230	91	1	1
	Single	21	7	0.09(0.13–0.69)	0.18(0.21–1.63)
	Divorced	10	9	2.275(0.89–5.78)	0.94(0.27–3.77)
	Windowed	11	15	4.212(1.78–9.97)	6.09(1.83–11.23)***
Education of the father	Unable to read and write	13	12	3.36(2.52–6.08)	0.46(0.91–2.27)
	Read and write	47	43	3.31(1.31–6.01)	1.38(0.48–4.09)
	Primary school	33	20	0.18(0.01–2.18)	0.79(0.26–2.38)
	Secondary school	61	23	2.59(1.31–5.17)	0.59(0.22–1.63)
	College and above	124	18	1	1
Occupation of the father	Government employee	134	25	1	1
	Farmer	7	14	3.72(1.93–9.22)	3.09(0.48–7.02)
	Daily labor	11	25	7.18(5.32–12.87)	3.61(0.92–6.12)
	Merchant	126	52	2.29(1.34–3.91)	1.33(0.52–3.41)
Education of the mother	Unable to read and write	8	7	3.37(1.55–7.32)	2.81(1.08–7.51)*
	Read and write	65	51	6.69(3.51–12.81)	2.28(0.93–5.57)
	Primary school	33	26	6.72(3.20-10.12)	0.87(o.45-4.32)
	Secondary school	43	17	1.37(0.37–3.51)	0.21(0.76–2.24)
	College and above	128	16	1	1
Share beds	Yes	204	104	3.14(1.64–6.05)	1.07(0.41–2.82)
	No	74	12	1	1
Share blades	Yes	60	83	7.14(5.57–14.98)	5.93(2.41–9.31)***
	No	218	33	1	1
Animal contact	Yes	207	96	1.65(0.95–2.86)	2.61(1.15–5.90)*
	No	71	20	1	1
Family similar illness	Yes	13	53	10.15(8.81–17.38)	8.49(3.73–11.39)***
	No	265	63	1	1
Family size	1–4	150	37	1	1
	5 and above	128	79	2.50(1.59–3.95)	1.69(0.79–3.62)
Living room	1–2	98	68	2.60(1.67–4.06)	2.71(1.42–5.14)***
	3 and above	180	48	1	1

## Discussion

The present study determined Tinea capitis status of school children in Gondar town. Finding from this study reveals a high prevalence of Tinea capitis, 29.4%with 95%CI (24.9-34.0%). This study is in line with study done in north east Iran(33.1%) [21] and study done in turkey28% [17, 22]. However, it is higher than study done in Ivory cost 13.9% [23]. The difference which might be due to the study done in Iverycost is supported by laboratory investigation of symptomatic children’s for Tinia capitis [24]. This might be due to the previous study done in Gondar town is the study participants only from public schools. Similarly, there is a time gap between this study versus the previous study conducted in Gondar town. In the meantime, there was the implementation of health education about hygiene by the town health office in the study area.

In this study, school children aged 5 to 8 years were near to four times more likely to have Tinea capitis as compared to those aged 12–15 this finding is supported by other local reports in the rural southern and north part of Ethiopia [25, 26]. This could be because they can’t take care of themselves at this age, and malnutrition is more prevalent in this age group than it is in the other age groups [27]. Furthermore, the function of hormonal factors in halting the development of dermatophytes [28, 29].

In our study, school children who were from public schools near to three times more likely to have Tinea capitis than school children who were from private schools. It might be due to most of the students in private schools being from high-income and more educated families as well as the presence of strict hygiene control in private schools than in public schools. The finding also showed that school children who were from sharing comb, blades, scissors, and towels at home were six times more likely to have Tinea capitis as compared to school children who have not shared this result supported by a study done in southwest Nigeria and Kenya [30, 31]. It’s the fact that fungus is contagious and often develops in young children. As well as it is usually spread by coming in contact with infected hairs on combs, brushes, towels, or pillowcases [32].

Our investigation showed that children with a history of animal contact were significantly higher than those who did not have contact with animals; this is compliant with a study done in Taiwan [33]. Children who live in fewer bedrooms are 2.7 times more exposed to Tinea capitis than children living in enough bedrooms. It might result in physical contact and contribute significantly in the illness’s dissemination. Furthermore, crowding highlights the vulnerability of inadequate personal hygiene and low socioeconomic standing [34, 35].

The likelihood of contracting Tinea capitis in children of widowed mothers was 6.1 times higher than in children of married mothers. It is predicted that married women can rely on their spouses for both financial support and emotional support when their child is ill; this helps them to share their responsibility for one another, which is essential to leading a healthy life, and to make solid decisions early on about seeking medical attention for their ill children [36]. However previous different scholars report that marital status was not associated with Tinea capitis among school-going children [18, 33, 37–40].

The likely hoods of being infected with Tinea capitis among children born to illiterate mothers were 2.8 times that of children born to highly educated mothers. This finding is similar to a study conducted in Chabahar, Iran [41]. A higher education level influences one’s ability to adopt a proactive attitude toward health, acquire important health-related knowledge, and correctly implement the many infection prevention and management strategies offered by healthcare professionals. Likewise, education is a source of income/wage which is crucial to lead a healthy life [42].

The likelihood of contracting Tinea capitis was 8.5 times higher in children whose family members also had the same illness than in other children., this finding is congruent with a study conducted in other parts of Ethiopia [18]. This might be due to sharing bedding and other personal items like combs and towels with those who are infected or through direct contact with the patient [37, 43]. Furthermore, Tinea capitis is highly contagious since it is often passed from person to person [44].

### Limitations of the study

The outcome variable determines by clinical sign and symptoms only it might affect the frequency of tinea capitis since this study does not employ fungal culture or microscopic examination and share the cross-sectional study drawback.

## Conclusion

The prevalence of tinea capitis was higher among schoolchildren in Gondar town. Young age, children from widowed marital status, illiterate mother, history of share blades, animal contact, a family similar illness, and lower number of living rooms are important factors contributing to tinea capitis among school children. Health education for the mother on the mode of transmission, prevention, and improve the low socioeconomic status of the parent is crucial.

## Data Availability

Data will be available upon request to the corresponding author.

## Abbreviations

SSA: Sub Saharan Africa
AOR: Adjusted Odds Ratio
CI: Confidence Interval
IRP: Institution Review Board
SPSS: Statistical Package for Social Science
